# Bio-Inspired Liquid-Cooled Plates for Enhanced Local Hotspot Dissipation in Lithium-Ion Battery Thermal Management

**DOI:** 10.3390/biomimetics11060432

**Published:** 2026-06-18

**Authors:** Xuguang Yang, Zhihui Wang, Xiaohua Gu, Yan Liu

**Affiliations:** 1 School of Energy and Building Environment, Guilin University of Aerospace Technology, Guilin 541004, China; 2 Engineering Research Center of Green Upgrade Key Technology for Energy Industry, Guilin University of Aerospace Technology, Guilin 541004, China; 3 State Key Laboratory for Modification of Chemical Fibers and Polymer Materials, College of Materials Science and Engineering, Donghua University, Shanghai 200051, China

**Keywords:** lithium-ion battery, battery thermal management, liquid-cooled plate, bio-inspired channel

## Abstract

To enhance the thermal management of lithium-ion batteries in new-energy vehicles, various bio-inspired liquid-cooled plate channel designs were investigated to improve hotspot dissipation within the laminar flow regime. A series of three-dimensional numerical simulations were conducted to compare leaf vein-, tree branch-, honeycomb-, and spider web-inspired channels, followed by further optimization to improve thermohydraulic performance. The selected optimized bio-inspired channels were subsequently evaluated against conventional structures. Simulation results indicate that the honeycomb-inspired liquid-cooled plate channel achieved the best performance, followed by the tree branch- and spider web-inspired channels, which exhibited comparable thermohydraulic performance. The leaf vein-inspired channel demonstrated the lowest performance. The key design element for enhanced heat dissipation is the inclusion of longitudinal branch channels, which minimize flow zones with near-zero velocity and effectively mitigate local hotspots. Furthermore, the combination of longitudinal and inclined branch channels can redirect flow direction and enhance fluid mixing. Compared with the conventional channel widely adopted in existing studies, within the Reynolds number range of 260 to 920, the optimized honeycomb-inspired liquid-cooled plate channel achieves a 44.0–49.3% increase in Nusselt number and an 81% enhancement in comprehensive performance metric. Concurrently, thermal resistance is diminished by 2.6–9.2%, and pumping power is reduced by 50.0–56.8%.

## 1. Introduction

The structural transformation of the global energy sector has accelerated the development of electric vehicles. Lithium-ion batteries have been extensively deployed as the primary power source due to their high energy density and extended driving range. However, the ideal operating temperature range for lithium-ion batteries is 20–40 °C. At elevated temperatures, lithium exhibits high chemical reactivity and becomes highly susceptible to thermal runaway and combustion. Moreover, short circuits may occur at high temperatures due to the complex internal architecture of lithium-ion battery packs [1]. Conversely, the lower temperature limit is equally critical in electric vehicle applications operating in cold climates, where active preheating is essential. In this context, thermally active materials such as induction-heating nanocomposites have shown promising potential [2]. Therefore, effective thermal management systems are indispensable for ensuring the safe and efficient operation of lithium-ion batteries across the entire temperature range.

Currently, liquid-cooled plate (LCP) is recognized as the most effective thermal management method, demonstrating superior thermal dissipation efficiency and lower manufacturing costs [3]. The critical determinant of its performance lies in the cooling channel configuration, which has become the central focus of academic research. In recent years, a series of innovative channel designs have been proposed to enhance the thermal performance of liquid-cooled plates. Notably, research on the cross-sectional aspect ratio of channels has yielded significant findings. For example, Yang et al. [4] designed a liquid-cooled plate with a small aspect ratio (*K* = 2), which yielded optimal thermal performance. Xu et al. [5] identified a suitable aspect ratio of 12.5 mm:1.6 mm for enhanced heat transfer efficiency. Ao et al. [6] investigated the thermohydraulic performance of microchannels with non-uniform aspect ratios, demonstrating improved flow distribution characteristics. Furthermore, Tian et al. [7] and Zhang et al. [8] innovatively designed sinusoidal corrugated channels, which exhibited superior heat transfer capabilities due to the induced secondary flows and increased effective heat transfer surface area. In addition, alternative channel configurations such as the three-step farmland-type side-recessed channels [9] and novel Tesla valve capillary channels [10] have also been recognized as promising liquid-cooled plate structures for enhancing cooling performance. 

Although these innovative designs can effectively enhance the performance of traditional LCPs, their optimization invariably suffers from the trade-off between heat transfer performance and power consumption. Multi-objective topology optimization methods can design unconventional channel structures that maintain steady battery temperatures without consuming significant power, representing a recent research hotspot [11,12,13,14]. For instance, Li et al. [15] employed a combination of experimental and simulation approaches to compare the temperature distributions between optimized straight channels and topology-optimized channels. The results demonstrated that the topology-optimized channels could achieve higher Nusselt numbers and Performance Evaluation Criteria while effectively mitigating hotspots. Ren et al. [16] found that compared with traditional straight channels, the topology-optimized channel reduced the average temperature, maximum temperature, and temperature gradient by 0.86 °C, 0.89 °C, and 1.12 °C, respectively. Sun et al. [17] developed a flow channel structure featuring multiple entrances and exits through topology optimization. Their optimization achieved a 2.53% reduction in the average temperature and a 57.67% reduction in the root mean square temperature of the heat source.

Unfortunately, innovative LCP channels are typically designed using empirical trial-and-error methods, which limits the achievement of significant performance improvements; in addition, topology optimization methods require extensive iterative computations, and from an engineering standpoint, the resulting complex structures significantly increase fabrication costs. Considering these limitations, researchers have increasingly turned to bio-inspired designs for LCP channel development [18,19].

Among these, designs based on the fractal structure of the branch are the main designs being created in current studies [20]. Specifically, Zhan et al. [21], Liu et al. [22], Chai et al. [23] and Xu et al. [24] introduced leaf vein-like branched channel configurations that maintained peak channel temperatures while achieving superior temperature uniformity compared with conventional straight channels. Alnaqi [25] developed a dendrite-inspired channel structure, demonstrating heat dissipation performance comparable to the biomimetic snowflake-type channel reported by Zhou et al. [26]. Furthermore, Wang et al. [27], Qi et al. [28] and Duan et al. [29] investigated Y-shaped channel architectures to optimize fractal dimensions, thereby enhancing the Performance Evaluation Criteria. Collectively, these studies have achieved significant improvements in heat transfer performance with only marginal increases in pressure drop.

Wang et al. [30] investigated a novel spider web-like cooling channel configuration to enhance fluid distribution and heat transfer uniformity, achieving Performance Evaluation Criteria of 1.27 and a uniformity index of 0.14. A similar spider web-like channel was proposed by another study [31], which increased the convective heat transfer coefficient by 35.2% while reducing pressure loss by 8.3%. Han et al. [32] designed a novel biomimetic petal-type channel to improve thermal performance and temperature uniformity; their results indicated that a five-inlet configuration demonstrated the best comprehensive heat transfer performance. Zhang et al. [33] further introduced a novel vascular flow channel architecture that improved heat dissipation while reducing pressure loss by 40.7%. Notably, a double-layer tree-like channel design from Fan et al. [34] reduced maximum temperature, surface temperature, and pressure drop by 1.79%, 69.25%, and 79.13%, respectively. Wang et al. [35] proposed a novel lemon-slice microchannel and optimized its geometric parameters, with the final configuration achieving a 4.24% temperature reduction and a 56.97% reduction in pressure drop. 

Additionally, fish-scale structures and surface modifications represent key aspects of biomimetic channel design, although comparatively fewer studies have focused on these configurations relative to fractal structures. For instance, Fan et al. [36] designed four fishbone channel configurations, optimizing them to achieve lower maximum temperatures, minimized temperature gradients, and reduced pressure drops. Wang et al. [37] and Tang et al. [38] installed biomimetic protrusion structures on channel surfaces to enhance heat transfer performance and achieve drag reduction.

Based on this literature review, the channel structure of an LCP has a significant impact on both its heat transfer performance and power consumption. However, the mechanisms by which bio-inspired channels enhance heat transfer while reducing flow resistance are still not well understood. Moreover, comparative studies of various bio-inspired LCP channels are lacking in public literature. In this study, a series of bio-inspired LCP channels are proposed, with leaf vein, tree branch, honeycomb, and spider web configurations used as the initial channel configurations. Furthermore, design optimization is conducted to further enhance the thermohydraulic performance. Finally, the flow and heat transfer performance under different Reynolds numbers is analyzed across the selected optimized channels and the conventional channel widely adopted in existing studies. The remainder of this paper is organized as follows: Section 2 describes the physical models for both the initial and optimized bio-inspired LCP channels, along with the numerical solution method and associated parameter definitions. Section 3 presents the model validation. Section 4 reports the numerical results and comparative analysis. Finally, Section 5 concludes the study and outlines directions for future research.

## 2. Problem Description

### 2.1. Physical Model

**Figure 1:**
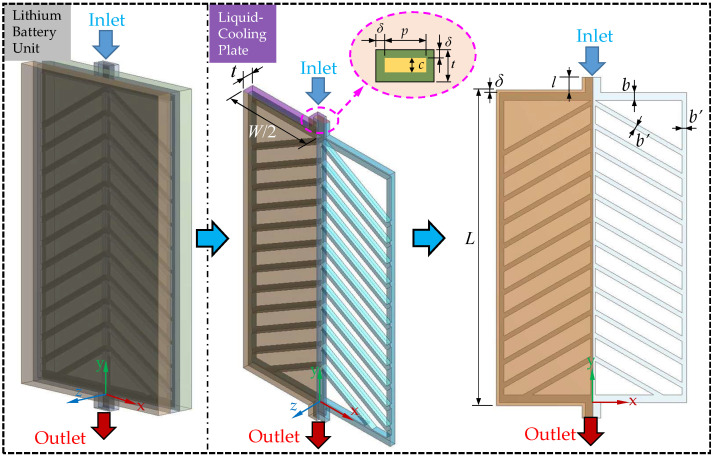
Schematic diagram of the liquid-cooled plate.

The numerical solution domain is illustrated in Figure 1. A 30Ah ternary polymer pouch lithium battery cell features a liquid-cooled plate structure in its central region. The LCP is characterized by a length *L*, width *W*, and thickness *t*. It is enclosed by a heat-conducting material shell with a wall thickness of *δ*. The geometric parameters in this work were kept consistent with those adopted in the published literature [21]. The cooling fluid enters through the inlet channel and exits from the outlet channel after traversing the main channel. The cross-sectional view denoted by the dotted lines indicates dimensions *p* and *c* for the length and width, respectively. The main channel assembly comprises distributed channels near the inlet and outlet, each with a width of *b*, connected to branch channels of width *b*′. Detailed geometric parameters of the LCP are listed in Table 1.

**Figure 2:**
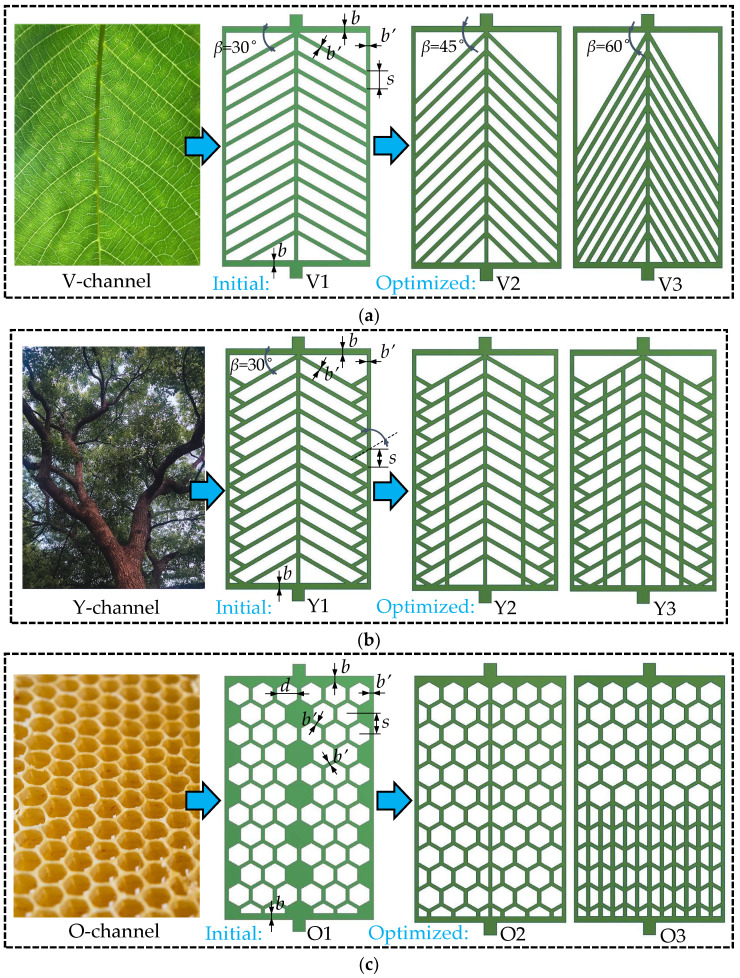

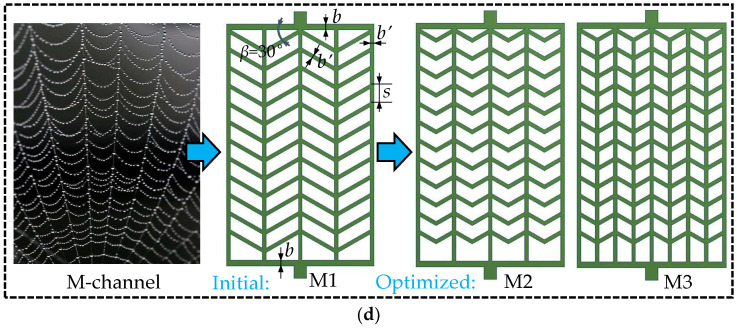
Schematic diagram of the bio-inspired liquid-cooled plate model: (**a**) V-channel, (**b**) Y- channel, (**c**) O-channel, (**d**) M-channel.

Nature employs various channel structures in numerous organisms to achieve efficient material transportation and energy conversion. Inspired by these biological systems, the present study proposes four bio-inspired liquid-cooled plate (BLCP) channel configurations, classified according to their geometric shapes as the V-channel, Y-channel, O-channel, and M-channel, as illustrated in Figure 2. The primary geometric parameters for these designs are defined as follows: *s* represents the spacing between adjacent branch channels, while *β* denotes the inclination angle of the inclined branches. For the O-channel, the side length of the hexagonal channel is represented by *d*. Detailed geometric parameters are provided in Table 2. 

For the V-channel series, the inclined branch configuration serves as the design basis. V1 represents the initial design with *β* = 30°, while the optimized configurations V2 (*β* = 45°) and V3 (*β* = 60°) incorporate progressively increased inclination angles. Similarly, the Y-channel series employs inclined branches in the initial configuration Y1. The optimized variants Y2 and Y3 integrate both longitudinal and inclined branch channels, differing in the number of longitudinal branches. A comparable design approach is applied to the O-channel and M-channel configurations. Notably, the hexagonal channel element integrates with four inclined branch channels and two longitudinal branch channels. Compared to the O1 channel, the O2 channel incorporates an increased number of both longitudinal and inclined branch channels. Similarly, the O3 channel features additional longitudinal branch channels relative to the O2 channel. For the M-channel series, the influence of the number of inclined branch channels on thermohydraulic performance is evaluated through comparison of the M1 and M2 channels, whereas the influence of the longitudinal branch channel count is assessed by comparing the M2 and M3 channels.

### 2.2. Governing Equations and Boundary Conditions

The three-dimensional, steady-state flow problem with constant fluid properties considered in this study was numerically solved using the Finite Volume Method. The numerical simulation is conducted utilizing the Ansys Fluent 19.2 software. The governing Navier–Stokes equations were resolved under the laminar flow assumption, with pressure-velocity coupling addressed by the SIMPLE algorithm. The corresponding governing flow equations are presented below [4]:

Continuous equation:

(1) $$\rho_{f}\nabla \cdot \left(\vec{U}\right)=0.$$

where *ρ*_f_ represents the density of the ordinary water and *U* represents the velocity vector of the coolant flow.

Momentum equation:

(2) $$\rho_{f}\frac{\partial \vec{U}}{\partial t}+\rho_{f}\left(\vec{U}\cdot \nabla \right)\vec{U}=-\nabla P+\mu \nabla^{2}\vec{U}.$$

Fluid energy equation:

(3) $$\rho_{f}C_{\mathrm{pf}}\frac{\partial T}{\partial t}+\rho_{f}C_{\mathrm{pf}}\left(\vec{U}\cdot \nabla T\right)=k_{f}\nabla^{2}T,$$

where *C*_pf_ denotes the specific heat capacity of the coolant, *T* represents the temperature, and *k*_f_ is the thermal conductivity of the fluid.

Solid energy equation:

(4) $$\rho_{s}C_{\mathrm{ps}}\frac{\partial T}{\partial t}=k_{s}\nabla^{2}T,$$

where *ρ*_s_ denotes the density of the LCP, *C*_ps_ represents the specific heat capacity of the solid, and *k*_s_ is the thermal conductivity of the solid. 

The boundary conditions of the numerical simulation are as follows [21]:

- Mass flow rate inlet:

(5) $$Q_{m}=0.005\mathrm{kg}/s,T=T_{\mathrm{in}\;}=298.15K.\;$$

- Pressure outlet:

(6) $$\;\frac{\partial u}{\partial z}=\frac{\partial v}{\partial z}=\frac{\partial w}{\partial z}=\frac{\partial T}{\partial z}=0.$$

- Constant heat flux surface of the LCP:

(7) $$u=v=w=0,q=2373.8{\;W/m}^{2}\mathrm{at}\;\mathrm{high}\;\mathrm{discharge}\;\mathrm{rates}.$$

To enhance computational efficiency, only half of the computational domain is simulated by exploiting geometric symmetry. Consequently, the symmetry plane is defined as a symmetrical boundary, while all other external surfaces are treated as adiabatic.

### 2.3. Parameter Definitions

The Reynolds number (*Re*) is expressed as follows:

(8) $$Re=\frac{\rho_{w}uD_{h}}{\mu_{w}},$$

where *μ*_w_ is dynamic viscosity, *u* is the flow rate and *D*_h_ is the hydraulic diameter.

The convective heat transfer coefficient (*h*) and the Nusselt number (*Nu*) are defined as follows:

(9) $$\left\{\begin{array}{l} h=\frac{Q}{A_{0}(T_{avg}-\frac{T_{\mathrm{in}}+T_{\mathrm{out}}}{2})},\\ Nu=\frac{h_{c}D_{h}}{\lambda_{w}}. \end{array}\right.$$

*T*_avg_ is the average temperature of the heat exchange surface on the LCP is defined as follows:

(10) $$T_{avg}=\frac{\int_{A}TdA}{\int_{A}dA},$$

where *A* represents the heat exchange area and *T* is the surface temperature.

To better understand the temperature uniformity of the LCP, the TUI (temperature uniformity index) is used as the evaluation index:

(11) $$\{\begin{array}{c} TUI=1-HF,\\ HF=\frac{T_{max}-T_{avg}}{T_{avg}}, \end{array}$$

where *HF* is the Heterogeneity Factor [39].

The thermal resistance of the LCP is defined as

(12) $$R_{\mathrm{th}}=\frac{T_{\max}-T_{\mathrm{in}}}{Q}.$$

The pressure drop (∆*p*) can be formulated as

(13) $$\Delta p=p_{\mathrm{in}}-p_{\mathrm{out}}.$$

A comprehensive performance merit (FOM) [21] is proposed to comprehensively consider both enhanced heat transfer and pump power consumption of the LCP.

(14) $$\left\{\begin{array}{l} FOM=\frac{h/h_{0}}{(P_{\mathrm{pump}}/P_{\mathrm{pump}0}{)}^{1/3}},\\ P_{\mathrm{pump}}=\Delta p\cdot Q_{v},\\ Q_{v}=\frac{Q_{m}}{\rho_{w}}. \end{array}\right.$$

where *P*_pump_ denotes the pump power consumption and *Q*_v_ and *Q*_m_ represent the volume and mass flow rates, respectively.

## 3. Validation of Numerical Results

### 3.1. Grid Independence Test

**Figure 3:**
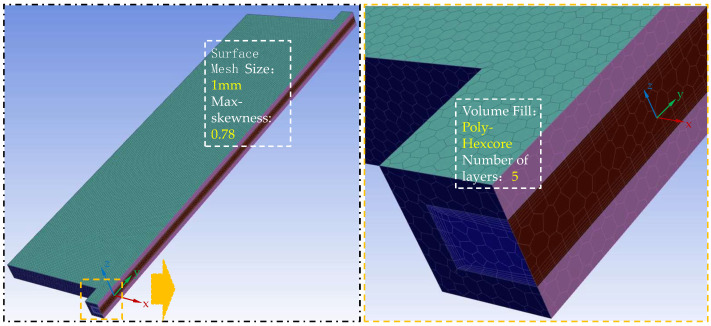
Polyhedral mesh strategy.

A grid independence study was conducted to verify that the numerical solutions were independent of the mesh size. Polyhedral cells, as illustrated in Figure 3, were employed to enhance mesh quality for complex geometries and improve computational efficiency. To maintain a y+ value close to 1, five layers of boundary layer meshes were applied to the fluid domain to ensure accurate resolution near the walls. The study employed varying numbers of grid cells: 68,055; 120,265; 181,527; 250,744; and 376,130. 

**Figure 4:**
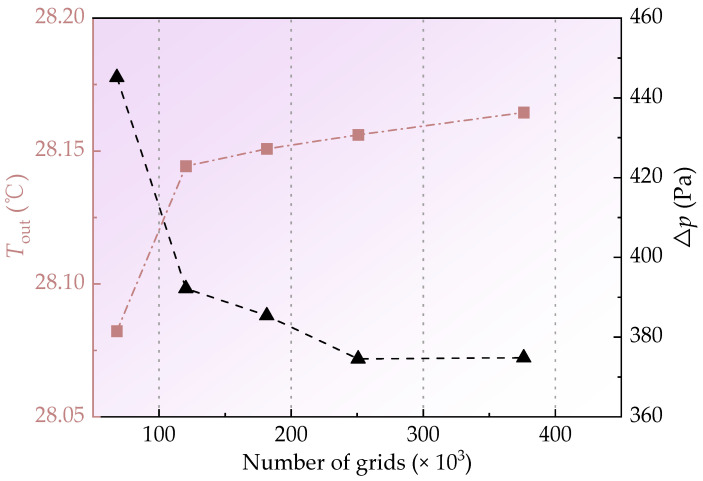
Grid independence test.

Figure 4 presents the variations in outlet temperature (*T*_out_) and pressure drop (∆*p*) with respect to the number of grid cells. As the number of grid cells increased, both the rates of change in *T*_out_ and ∆*p* became minimal. The results show that the relative errors of *T*_out_ and ∆*p* between the 250,744-cell and 376,130-cell meshes are only 0.03% and 0.08% (less than 0.1%), respectively. Balancing computational accuracy with efficiency, a mesh containing 250,744 cells was selected for the subsequent simulations.

### 3.2. Model Validation

**Figure 5:**
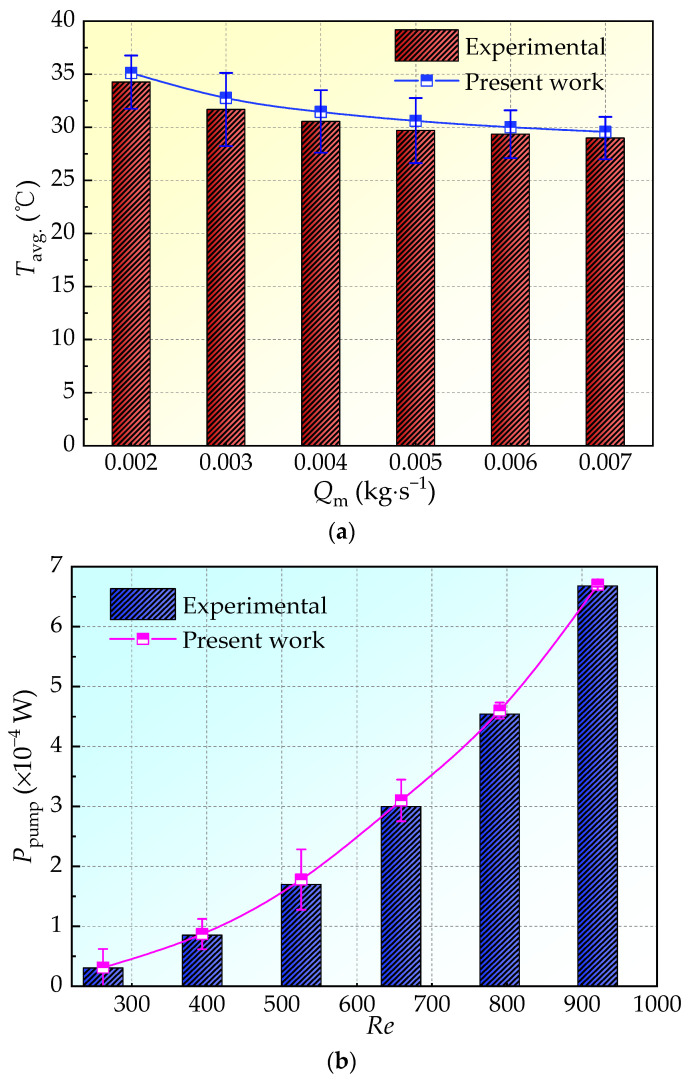
Comparison of results of simulation and experiment data: (**a**) *T*_avg_, (**b**) *P*_pump_.

The validity of the numerical model was verified by comparing the simulation results with the experimental data from Ref. [21], as shown in Figure 5. As illustrated in the figure, the inlet mass flow rate (*Q*_m_) varied from 0.002 to 0.007 kg/s, resulting in corresponding variations in the average temperature (*T*_avg_) that align well with those reported in Ref. [21]. The maximum deviation in the *T*_avg_ is 3.44% at *Q*_m_ = 0.003 kg/s. Figure 5b presents the relationship between pump power consumption (*P*_pump_) and Reynolds number (*Re*), where *Re* varies from 260 to 920. As shown in the figure, the numerical and experimental results are in good agreement, and the maximum deviation of 5.1% occurs at *Re* = 525. Overall, the numerical results demonstrate excellent agreement with the experimental data, confirming the high reliability of the proposed model for subsequent investigations. 

## 4. Results and Discussion

### 4.1. Numerical Results of Various BLCP Channels

**Figure 6:**
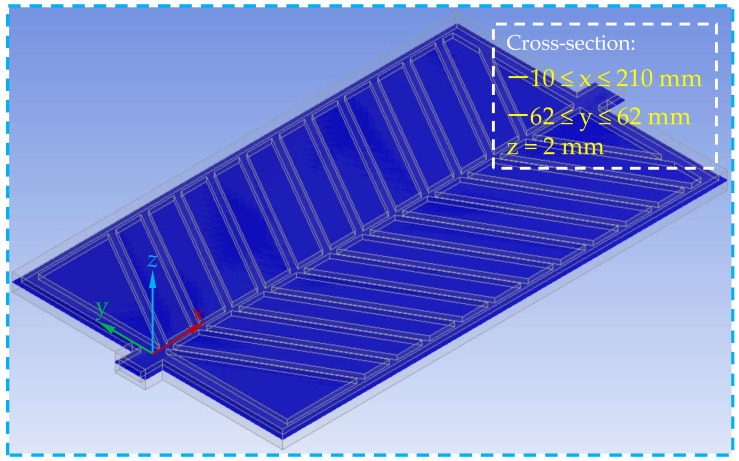
z = 2 mm section position.

The thermohydraulic performance of the BLCP channels is thoroughly analyzed by examining the temperature, pressure, and velocity distributions within the flow channels. Figure 6. illustrates the position of the z = 2 mm cross-section in the BLCP channel, which is the centre of the channel. 

**Figure 7:**
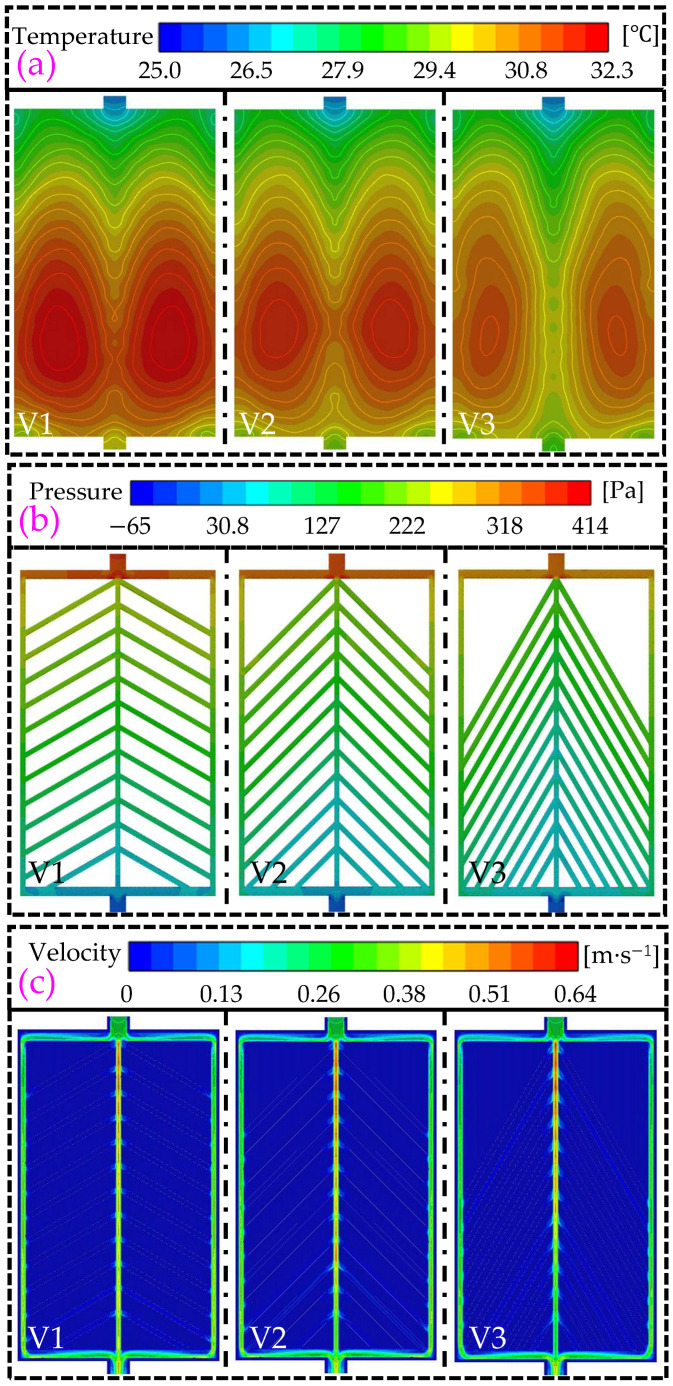
Contour distributions of the V-channels: (**a**) temperature, (**b**) pressure, (**c**) velocity.

Figure 7 illustrates the temperature, pressure, and velocity distributions of the V-channels at the z = 2 mm cross-section. It is observed that the temperature near the inlet is relatively low, whereas most of the heat is concentrated near the outlet, where the temperature exceeds 32 °C. Figure 7a shows the temperature contours exhibit elevated values along both channel sides with a lower temperature in the central region. This is primarily because the longitudinal branch channels are mounted in the central region, which effectively decreases the surface temperature. As the inclination angle *β* increases, both the hotspot area and the maximum surface temperature *T*_max_ decrease, indicating that branch channels with larger *β* are more conducive to heat dissipation. 

Furthermore, as shown in Figure 7b, a larger inclination angle *β* can effectively reduce the pressure drop. The maximum pressure occurs at the inlet and distribution channels, whereas inclined branch channels with a larger *β* contribute to minimizing the overall pressure drop across the channel. However, as shown in the velocity contours, the flow zones with near-zero velocity are more extensive, and regions of higher velocity are mainly observed at the longitudinal branch in the middle and both sides of the channel. This phenomenon occurs because the coolant enters through the inlet and flows sequentially through the middle channel and the side channels. Due to the narrow cross-section of the middle channel, the flow velocity reaches approximately 0.64 m/s, which necessitates greater pumping power. Consequently, the symmetrically distributed branch channels cannot be adequately supplied with coolant in a timely manner, impeding efficient heat removal. As a result, the highest temperatures are concentrated near the outlet on both sides.

**Figure 8:**
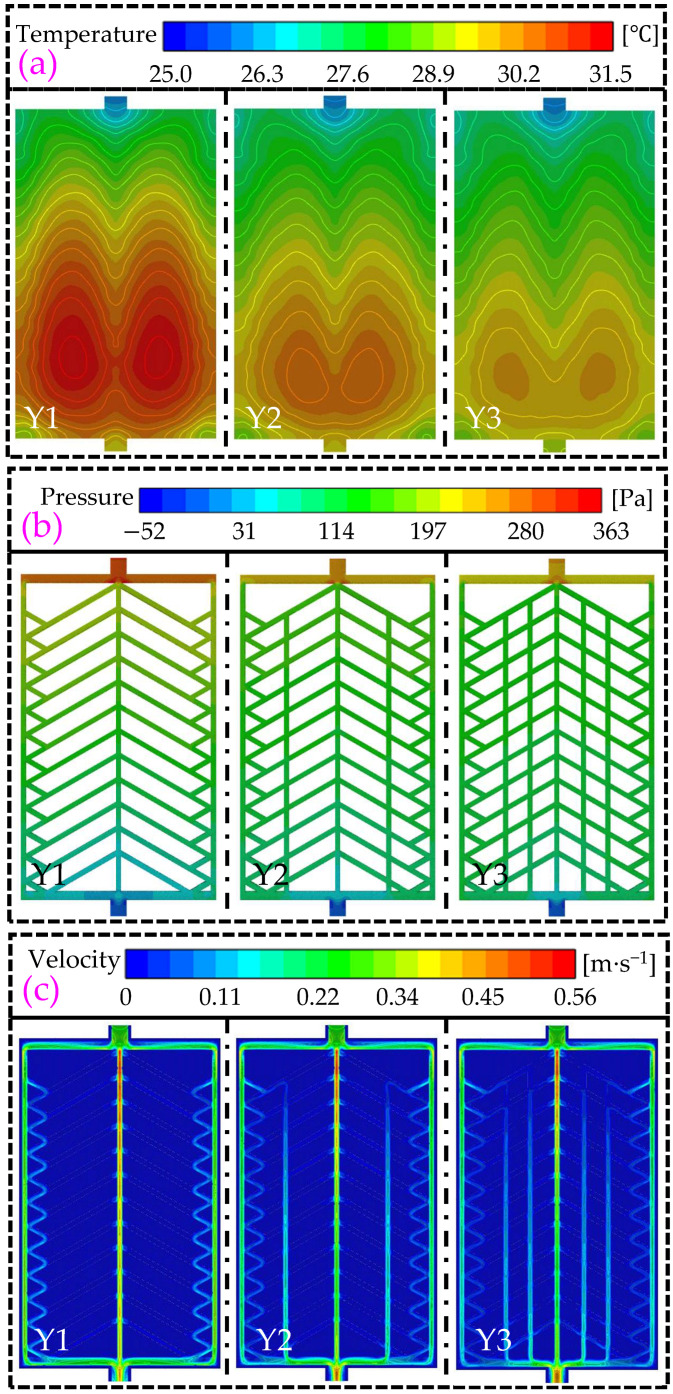
Contour distributions of the Y-channels: (**a**) temperature, (**b**) pressure, (**c**) velocity.

Figure 8 depicts the contour distributions of the Y-channels at the same z = 2 mm cross-section. The results indicate that in the Y-channels, high-temperature regions persist near both sides of the outlet, although the maximum temperature (*T*_max_) has slightly decreased. Notably, as the number of longitudinal branch channels increases, the hotspot area significantly diminishes, confirming that the longitudinal branch channels effectively reduce high-temperature regions. Furthermore, the overall temperature uniformity across the cooling plate is improved.

Additionally, Figure 8b shows that the pressure drop is significantly reduced. The maximum pressure distribution also occurs at the inlet and distribution channels; however, the pressure drop is noticeably reduced as the number of longitudinal branch channels increases. In contrast to the V-channels, the Y-channels utilize multiple branched channels for coolant entry, which reduces pumping power requirements and limits the maximum velocity to 0.56 m/s. Compared to the V1 channel, the Y1 channel features fewer inclined branch channels mounted on both sides, which enhance the high-velocity zones to reduce the hotspot area. In contrast, the optimized Y2 and Y3 channels incorporate additional longitudinal branch channels to further minimize the flow zones with near-zero velocity, as illustrated in Figure 8c.

**Figure 9:**
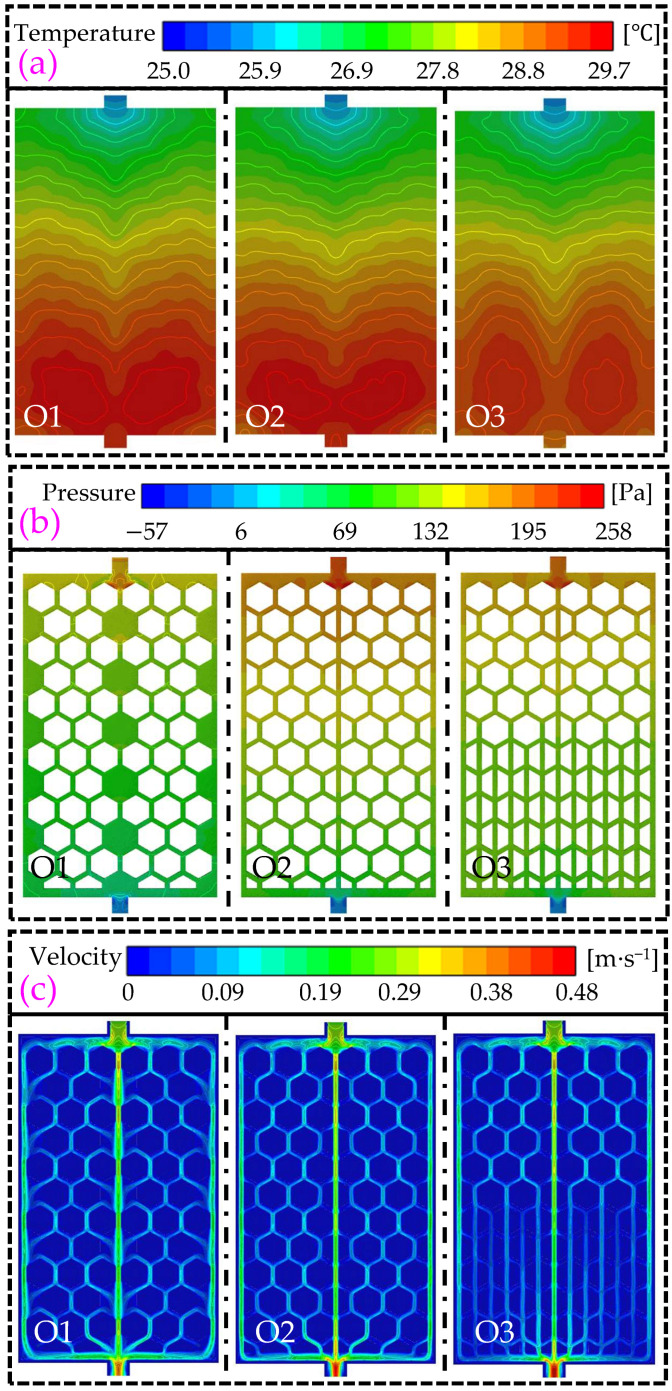
Contour distributions of the O-channels: (**a**) temperature, (**b**) pressure, (**c**) velocity.

**Figure 10:**
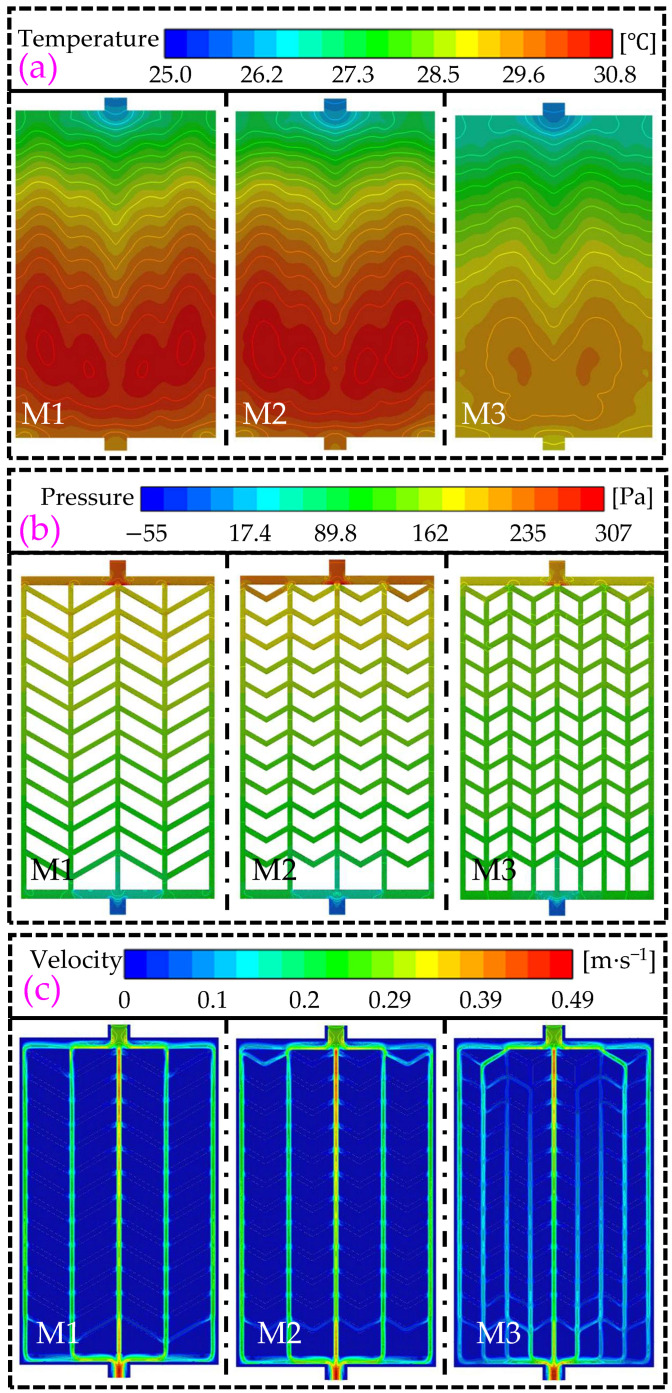
Contour distributions of the M-channels: (**a**) temperature, (**b**) pressure, (**c**) velocity.

The temperature contour distributions for the O-channels and M-channels are illustrated in Figure 9a and Figure 10a, respectively. Both the maximum temperature and temperature uniformity have significantly improved. In terms of maximum temperature (*T*_max_), the O-channels exhibited superior thermal performance. Relative to the V-channels, *T*_max_ was reduced by 2.6 °C, while the M-channels achieved a reduction of 1.5 °C. Notably, the phenomenon of elevated temperature contours at both channel sides with lower temperatures in the central region was effectively mitigated, resulting in a lower temperature distribution at the inlet and higher temperatures toward the outlet. This improvement stems from the combined presence of longitudinal and inclined branch channels in both the O-channels and M-channels, which enhance heat dissipation through switched flow direction and improved fluid mixing.

The pressure contour distributions for the above two types of channels are presented in Figure 9b and Figure 10b. It is observed that the pressure drop values are lower than those of the V-channels and Y-channels. Among the O-channels, the initial O1 configuration achieves the lowest pressure drop, whereas for the M-channels, the M3 configuration is preferred as the primary selection. Taking the M-channel series as a representative case, compared with the M1 and M2 configurations, the addition of inclined branch channels maintains an essentially unchanged pressure contour distribution. However, when comparing the M2 and M3 configurations, the addition of longitudinal branch channels effectively reduces the pressure difference.

Figure 9c and Figure 10c illustrate that the insufficient flow issue on both sides of the V- and Y-channels, which hindered timely heat removal, has been effectively addressed in the O- and M-channels. Upon entering the BLCP, the coolant flows through complex branching channels and is evenly distributed to the symmetrical side channels. Consequently, the coolant velocity across the entire BLCP achieves high uniformity, substantially reducing the temperature standard deviation within the plate. Moreover, this design significantly lowers pumping power consumption compared with the V- and Y-channels. The maximum velocity (*V*_max_) is comparable for O-channels and M-channels, notably, the *V*_max_ values of these channels are lower than those observed in V-channels and Y-channels. Channel velocity is strongly coupled with pressure drop, meaning that a lower *V*_max_ corresponds to a lower pressure drop. Furthermore, heat transfer performance is influenced by flow velocity within the channels. In O-channels, a significant high-velocity region is established. Interestingly, smaller flow zones with near-zero velocity are present, which enhance heat transfer efficiency by effectively mitigating local hotspots. A similar phenomenon is observed in the M3 channel, which demonstrates the best heat transfer performance among the entire M-channel series.

Table 3 presents the numerical results for all BLCP channels, including the maximum temperature (*T*_max_), temperature uniformity index (TUI), pressure drop (Δ*p*) and maximum velocity (*V*_max_). It is observed that the V-channel series exhibit relatively high *T*_max_ values, with the initial V1 channel reaching 32.28 °C, which ranks the highest among all channels. The Y-channel and M-channel series display similar *T*_max_ values, whereas the lowest *T*_max_ is achieved at the O-channel series. The maximum temperature uniformity index (TUI) corresponds to the V3, Y3, O3, and M3 channels within their respective series, with the Y3 channel achieving the best temperature uniformity. Notably, compared to the initial V1 channel, the optimized O3 channel demonstrates a decrease of 2.88 °C in maximum temperature (*T*_max_) and an increase of 0.0164 in the temperature uniformity index (TUI), indicating significant optimization across all performance indicators. In terms of pressure drop, the maximum ∆*p* occurs in the initial V1 channel at 374.87 Pa, which is 50% higher than the initial O1 channel exhibiting the minimum ∆*p*. Notably, despite the larger pressure drop yielding higher flow velocity, the maximum velocities (*V*_max_) for the M2 channel remains lower than those for the Y3 channel, indicating a non-linear relationship between pressure and velocity distributions. Based on comprehensive evaluation, the V3, Y3, O3, and M3 channels are selected as the optimal BLCP designs, demonstrating superior performance compared to conventional structures across a wide range of Reynolds numbers.

### 4.2. Comparisons with Other LCP Channels

**Figure 11:**
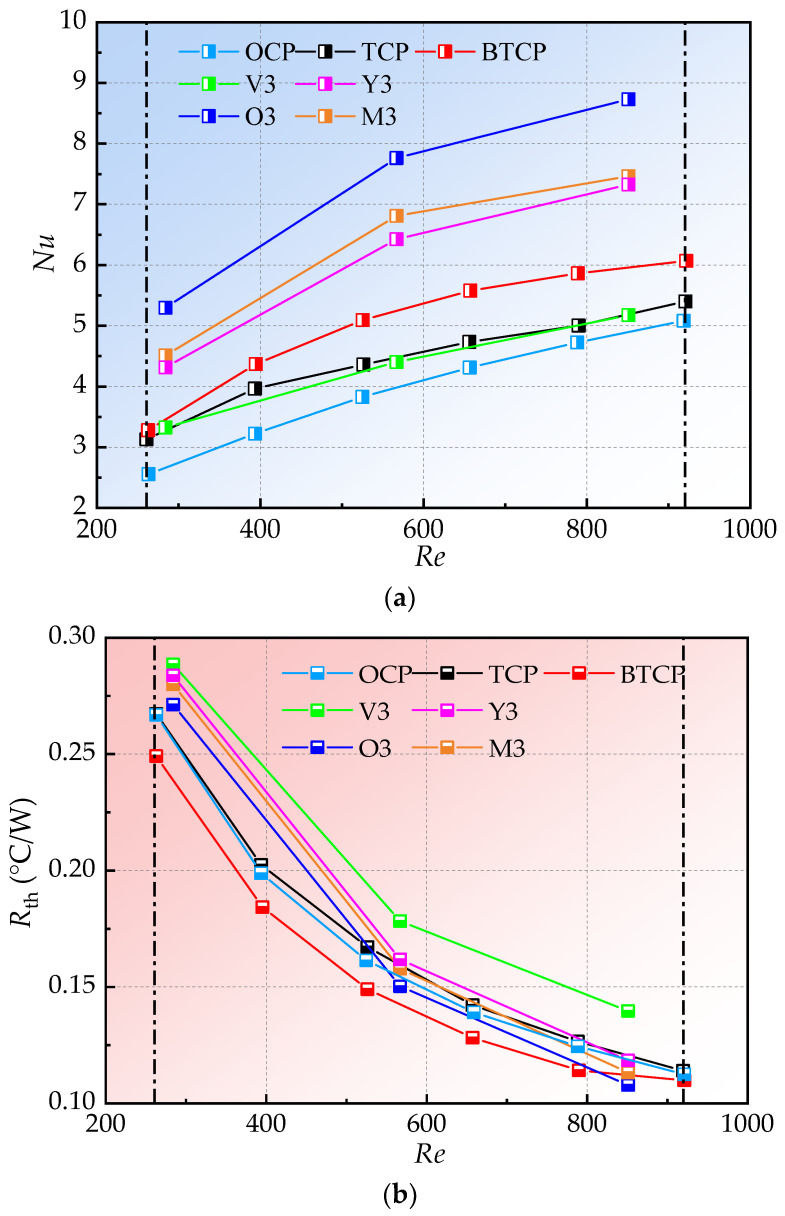
Comparison of the heat transfer performance: (**a**) *Nu*, (**b**) *R*_th_.

Figure 11 presents a comparative analysis of the heat transfer performance between the selected BLCP channels and those reported in the literature. The structures evaluated in previous studies [21] include the original channel (OCP) and two topology-optimized channels (TCP and BTCP). Figure 11a illustrates the variation in the Nusselt number (*Nu*) and thermal resistance (*R*_th_) with the Reynolds number (*Re*) to quantify heat transfer enhancement. It is evident that *Nu* exhibits an increasing trend with increasing *Re* for all channels. Notably, the O3 channel demonstrates significantly enhanced performance compared with the other channels. Relative to the OCP channel, *Nu* increases by 44.0% to 49.3%. The M3 and Y3 channels achieve comparable performance while still exhibiting higher *Nu* values than the channels reported in the literature. Among the selected optimal BLCP channels, the V3 channel exhibits the lowest heat transfer performance, which is similar to that of the TCP channel. Figure 11b shows a decreasing trend of *R*_th_ with increasing *Re*. The O3, Y3, and M3 channels demonstrate comparable performance to the channels in the literature, with the performance gap becoming more pronounced as *Re* increases. Notably, at *Re* > 600, the O3 channel exhibits lower *R*_th_ values compared to both the OCP and TCP channels. Relative to the OCP channel, *R*_th_ is reduced by 2.6% to 9.2%.

**Figure 12:**
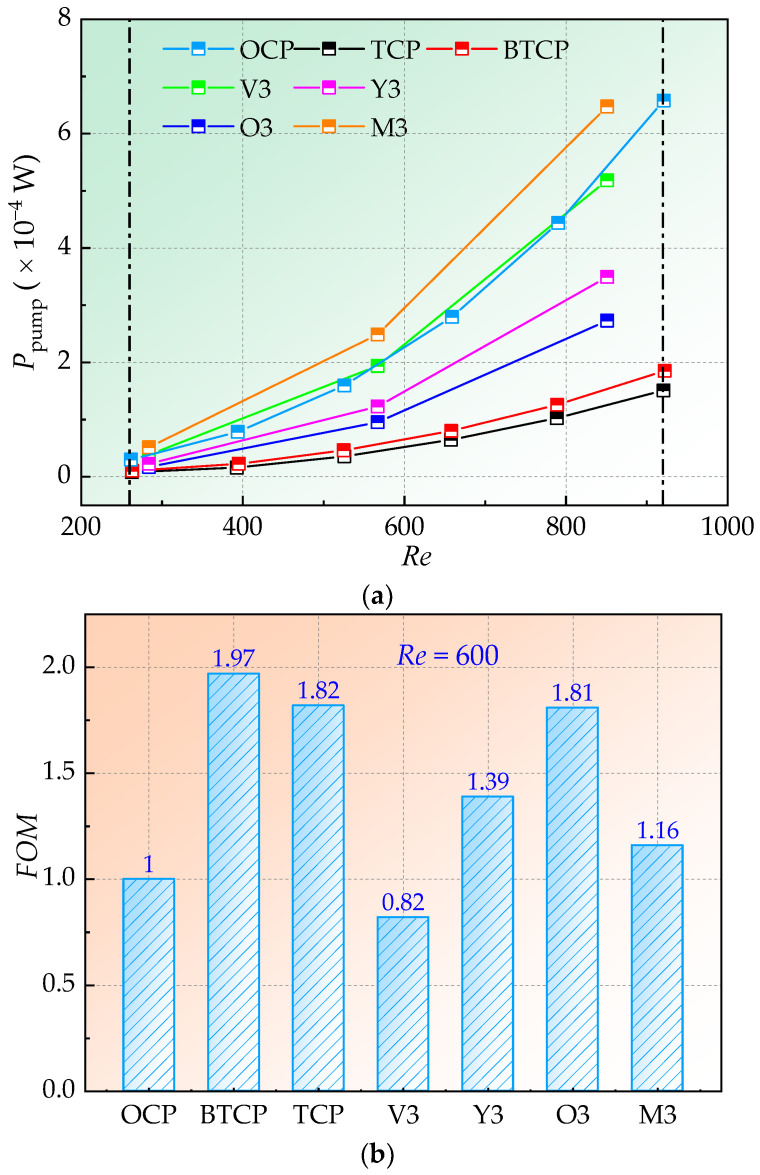
Comparison of power consumption and comprehensive performance: (**a**) *P*_pump_, (**b**) *FOM*.

As illustrated in Figure 12a, the variation trend of the pump power (*P*_pump_) across the BLCP channels is consistent with that of the Nusselt number (*Nu*). Both the O3 and Y3 channels exhibit lower *P*_pump_ values compared to all other selected optimal BLCP channels, with magnitudes remaining below those of the OCP channel. The V3 channel demonstrates a trend similar to that of the OCP channel, which serves to further validate the accuracy of the present numerical solution. Specifically, when compared to the OCP channel, the *P*_pump_ of the O3 channel reduces by 50.0% to 56.8%.

To balance the heat transfer performance and power consumption of the LCP channels, the comprehensive performance index (*FOM*) as a function of *Re* was introduced, as shown in Figure 12b, where the OCP channel is normalized as *FOM* = 1. As evident from the results, the *FOM* of the V3 channel is less than 1, whereas all other selected optimal BLCP channels demonstrate *FOM* values exceeding 1. Notably, the O3 channel achieves the highest *FOM* value, which is 81% higher than that of the OCP channel. The Y3 channel ranks second, achieving a 39% improvement, while the M3 channel shows a 16% increase. Importantly, the O3 channel exhibits performance comparable to the topology-optimized channels (TCP), which demonstrates that the bio-inspired liquid-cooled plate channel design can achieve the same effectiveness as topology-optimized designs.

## 5. Conclusions

In the present study, leaf vein-, tree branch-, honeycomb-, and spider web-inspired LCP channels were proposed to enhance heat transfer performance and reduce power consumption in the thermal management of lithium-ion batteries. Both initial designs and further optimized configurations were developed to improve the thermohydraulic performance. The selected BLCP channels (V3, Y3, O3, M3), which demonstrated superior performance in their respective categories, were compared with conventional structures across a wide range of Reynolds numbers. Based on the comparative analysis of numerical results, the primary findings are as follows:

(1) Based on the flow and heat transfer contour diagrams, the O-channels obtained the best flow and heat transfer performance, followed by the Y-channels and M-channels, which demonstrated similar performance. The V-channels exhibited the lowest performance among all configurations. Notably, the key design feature of the optimal configurations—specifically, the longitudinal branch channels—exerts a significant influence on flow and heat transfer performance. Compared to the initial V1 channel, the optimized O3 channel demonstrates a decrease of 2.88 °C in maximum temperature, an increase of 0.0164 in the temperature uniformity index and a reduction of 162.76 Pa in pressure drop.
(2) The enhanced heat transfer performance mechanism of the longitudinal branch channels eliminates flow zones with near-zero velocity, which effectively mitigates local hotspots. Furthermore, only the inclined branch channels can increase the pressure drop and maximum velocity, leading to degraded heat dissipation. However, the combination of longitudinal and inclined branch channels can redirect the flow direction and improve fluid mixing, thereby refining the thermohydraulic performance of the channel.
(3) At a larger Reynolds number range, compared to the OCP channel, which is the conventional channel widely used in existing liquid-cooled plate studies, the Nusselt number (*Nu*) and comprehensive evaluation metric (*FOM*) of the O3 channel increased by over 44% and 80%, respectively. Furthermore, the thermal resistance (*R*_th_) and pump power (*P*_pump_) decreased by over 2.6% and 50%, respectively. Additionally, when compared to other topology-optimized channels (TOC), the *FOM* of the O3 channel demonstrates comparable performance.
(4) Overall, the present numerical results confirmed the existence of optimal bio-inspired configurations for LCP channels, although the study has some limitations. First, the numerical model was validated against experimental data from the literature rather than through dedicated experimental validation conducted by the authors; second, comparing the performance of different geometries in the design of LCP was restricted to laminar flow, constant fluid properties, and steady-state conditions. These limitations constrained the scope of the current findings and defined the research focus for future work on developing high-efficiency BLCP channels.

## 6. Future Research

In future work, we will investigate the effect of various BLCP channels on heat transfer performance and power consumption using an experimental approach. Additionally, optimization algorithms will be employed to determine the optimal structural parameters for the enhanced O3 channel design.

## Figures and Tables

**Table 1:** Geometric dimensions of liquid-cooled plate.

Parameters	Symbol	Value/mm
LCP length	*L*	200
LCP width	*W*	124
LCP thickness	*t*	8
Distributed channel width	*b*	5
Branch channel width	*B*′	3
Shell thickness	*δ*	2
Cross-sectional length	*p*	10
Cross-sectional width	*c*	4

**Table 2:** Dimensional details of bio-inspired liquid-cooled plate.

Parameters	Symbol	Value/mm	Type
Spacing	*s*	15	V1-3; Y1-3; M1-3
		16.5	O1-3
Inclination angle	*β*	30°	V1; Y1-3; M1-3
		45°	V2
		60°	V3
Hexagonal length	*d*	8.5	O1-3

**Table 3:** Results of numerical solution for all BLCP channels.

Type		*T*_max_/°C	TUI × 10^2^	∆p/Pa	*V*_max_/m_/_s
V-channel	V1	32.28	94.57	374.87	0.64
	V2	31.91	95.06	363.20	0.61
	V3	31.44	96.00	343.66	0.58
Y-channel	Y1	31.41	95.96	326.86	0.56
	Y2	30.51	95.79	280.37	0.56
	Y3	29.95	96.35	267.47	0.55
O-channel	O1	29.68	95.72	187.38	0.40
	O2	29.60	95.56	226.20	0.47
	O3	29.40	96.21	212.11	0.46
M-channel	M1	30.69	94.55	265.67	0.47
	M2	30.76	94.98	268.47	0.49
	M3	29.68	95.58	227.75	0.46

## Data Availability

All data supporting the results of this study are included in this article.
